# Nephrolithiasis, Nephrotomy

**Published:** 1880-10

**Authors:** Herman Mynter


### NEPHROLITHIASIS, NEPHROTOMY.

BY HERMAN MYNTER, M. D.

Mrs. D., 45 years of age, of a healthy family, never had any serious disease except smallpox when 15 years of age. She has borne eleven children in normal deliveries. During the pregnancies she has always complained of burning on urinating, and of pains of doubtful character in the abdomen. During her last pregnancy, two and a half years ago, the urine had for four weeks a reddish color, the pains were more severe, and she kept her bed for four weeks. She has never discovered gravel or stone in the urine.

Her disease commenced in August, 1878, with pains in the left lumbar region. These increased gradually and were considered to be of rheumatic origin, and were consequently treated with liniments and cataplasma, with relief for a time. About Christmas, 1878, the pains increased again, and soon became severe. For three months subsequently she was confined to her bed, and for six weeks constant injections of morphine were resorted to. She had for some time considerable fever (104 to 105° Fahr.) In January, 1879, a tumor had been discovered in the lumbar region, which slowly increased and extended forwards and upward in the region of the spleen. She had then constant pain and burning on micturation, but the chemical examination of the urine proved normal. In March, 1879, her physician, Dr. Tobie, aspirated the tumor from the lumbar region, and evacuated about 4 ℥ matter, with considerable relief to the patient. The swelling increased again and perforated at last at the point of puncture in May, 1879, about one pint of matter being evacuated. She felt considerably better for a long time, and the fistula discharged freely. In September a mass several inches long and half inch thick, of firm consistency and whitish color, but whose nature was not ascertained, was discharged through the fistula, followed by a copious evacuation of matter, about one pint in all. In December a similar, but smaller piece, was discharged, but the pains increased again, and she was again obliged to keep her bed. As long as the matter discharged freely through the fistula, the patient felt comfortable, but if the discharge stopped, the pains and difficulties in urinating increased, and the urine contained pus in considerable quantity. She was sent to me by Dr. Tobie for surgical treatment December 20th, 1879, at which time the condition was as follows: A swelling was felt along the whole left lumbar region, extending in front into the vicinity of the spleen, where a tumor was perceived, much similar to an enlarged spleen, but not reaching above the lower margin of the ribs. The tumor was of a solid consistency and not fluctuating. The twelfth rib was considerably nearer the crist of the ileum than on the healthy side. Midway between the end of the twelfth rib and crista ilii there was found a fistulous opening, through which a sound could pass three inches upwards, inwards and to the right. No denuded bones or stones could be felt. The fistula was discharging quite freely at this time, and she, therefore, felt comfortable. An examination of three samples of urine, taken eight hours apart, showed two (midday and evening) to be clear, without albumen and with but a few pus corpuscles, while the third

(morning) contained a considerable residium, which under the microscope consisted of innumerable fine pus corpuscles, a few epithelial cells from the bladder and no casts. This sample contained considerable albumen.

Dec. 30th. Two stones of considerable size were discharged through the fistula to-day, thus fixing the diagnosis to nephrolithiasis.

After consultation with Dr. Miner, operation was determined upon, with the view of extirpating the kidney, and performed Dec. 31st under chloroform, Drs. Miner, Tobie, Hauenstein, Lothrop and Diehl being present. An incision was made from the eleventh rib to crista ilei through the fistula, but no trace was left of the normal anatomy. The whole tissue between twelfth rib and the crest of the ileum consisted of fibrous, unyielding almost cartilaginous tissue, which was divided with difficulty. To give more place, two inches of the eleventh rib were resected, subperiostally as in Czerny's method for extirpation of the kidney, but without much advantage resulting. Fascia lumbo-dorsalis, the sacrospinalis and quadratus lumborum muscles were all included in, and transformed into this fibrous tissue which was about three inches thick. By dilating and following the fistula the finger at last reached the place of the kidney, but the kidney itself could not be distinguished nor isolated, the finger meeting the same hard tissue everywhere. The margin of the quadratus lumborum muscle was the only thing, which could positively be recognized. By following the fistula farther downward, a little cavity was found filled with gravel and stones, which were removed. They consisted of phosphates and weighed about 2 z. The fistula in its whole length was filled with soft bleeding granulations. As it was impossible to isolate and extirpate the kidney, the cavity was syringed out, with carbolized water (5 per cent.), and covered with a solution of carbolic acid and sweet oil (1 to 10), and salicylic jute, a drainage tube having been introduced. The farther course was favorable for a time. The wound healed, except where the drainage tube was left, the tumor in front got smaller and visibly retracted, the appetite increased, everything seemed to promise a good result, when on January 20th, 1880, a crupous pneumonia set in, which terminated fatally Jan. 24th.

At the post-mortem examination the whole left lung was found in a state of hepatization from pneumonia. In the abdominal cavity a tumor was seen between colon descendens and the mesenterium of ileum, pressing the peritoneum forward and the descending colon outward. The tumor was as large as the fist, fluctuating on top, but with that exception, hard as cartilage, adherent to the pancreas and colon descendens. The fluctuating part was incised and contained pus, and stones of different sizes. The whole tumor was extirpated, in connection with parts of the organs, to which it was adherent, it being impossible to separate them.

The tumor consisted of the kidney, which was transformed into a retention cyst, containing a great number of cavities, all connected with the pelvis of the kidney, and containing any number of stones and much broken down pus. No trace of the normal kidney tissue was left, that organ being replaced by this sac with hard cartilaginous walls and septa. The incision had opened into what was the pelvis of the kidney.

This case is of interest, as showing that there is, or may be, a limit in which the extirpation of the kidney is justifiable, even in cases of stones in the kidney; for we see that long continued suppuration can produce such changes in the organ itself and the surrounding tissues, as to make the extirpation virtually impossible.

